# Distinct stress, eating, and body image phenotypes among university students: a latent class analysis in Saudi Arabia

**DOI:** 10.3389/fpubh.2026.1822639

**Published:** 2026-07-02

**Authors:** Ahmed Alabdrabalnabi, Najla M. Aljehani

**Affiliations:** 1 Department of Public Health, College of Health Sciences, Saudi Electronic University, Dammam, Saudi Arabia; 2 Department of Public Health, College of Health Sciences, Saudi Electronic University, Riyadh, Saudi Arabia

**Keywords:** body dissatisfaction, feeding and eating disorders, psychological, Saudi Arabia, stress, students

## Abstract

**Introduction:**

Disordered eating and body image disturbances are increasingly recognized as pressing public health concerns among young adults. While variable-centered approaches dominate the literature, they may obscure heterogeneity by assuming uniform associations between stress, body image, and eating behaviors. In Saudi Arabia, where rapid sociocultural change, Western beauty ideals, and rising obesity intersect with unique cultural values and academic pressures, university students may exhibit distinct psychosocial phenotypes. Therefore, the study applied latent class analysis (LCA) to identify distinct subgroups characterized by varying levels of stress, body image dissatisfaction, and disordered eating attitudes among students at the Saudi Electronic University.

**Methods:**

A cross-sectional online survey was conducted from February to March, 2025 across 15 campuses of the Saudi Electronic University. Validated instruments included the Perceived Stress Scale (PSS-10), Eating Attitudes Test (EAT-26), and Body Shape Questionnaire (BSQ-8). Latent class models (2–5 classes) were estimated using Gaussian mixture modeling. Bayesian Information Criterion, model parsimony, and interpretability guided class selection. Demographic correlates (age, sex, BMI, marital status) were assessed using χ^2^, ANOVA, and multinomial logistic regression.

**Results:**

Among 771 respondents (mean age 25·1 years, 69.65% women, mean BMI 24.94 kg/m^2^), a four-class solution best fit the data. Class 0 (22·8%) exhibited Body Image Concerned –High Eating Concerns; Class 1 (30·7%) high stress with severe body-image dissatisfaction; Class 2 (9·9%) high eating concerns but low body-image dissatisfaction; and Class 3 (36·6%) moderate stress and body-image vulnerable with modest eating attitudes. Higher BMI increased the odds of Class 0 but reduced the odds of Classes 1 and 3. Female sex predicted lower odds of Classes 1 and 3 but a tendency toward Class 2. Age and marital status were not consistent predictors.

**Conclusion:**

Saudi university students exhibit heterogeneous stress–eating–body image profiles, including a large subclinical group and a behaviorally at-risk subgroup without overt body-image dissatisfaction. These findings support stratified prevention strategies: stress-management for stress-dominated groups, behavioral monitoring for Class 2, and early preventive interventions for Class 3. Culturally adapted, sex-sensitive approaches aligned with Saudi Vision 2030 are warranted to reduce the burden of disordered eating.

## Introduction

Disordered eating behaviors and body image disturbances are increasingly recognized as pressing public health concerns globally, particularly among young adults (1–3). Negative body image has been consistently associated with maladaptive eating patterns, psychiatric morbidity, and poorer psychosocial outcomes (4). On the other hand, positive body image may function as a protective factor, promoting healthier relationships with food and adaptive self-regulation (5). Importantly, evidence suggests that positive and negative body image are not opposite ends of a continuum but qualitatively distinct constructs that may coexist, underscoring the complexity of body image–related experiences (6, 7). This recognition highlights the need for multidimensional approaches to understand how these constructs interact with eating behaviors and stress, rather than focusing on single isolated factors.

The Embodied Self Model provides a conceptual framework for these dynamics (8, 9). It suggests that being in tune with internal signals of hunger, fullness, and emotional states fosters adaptive eating, whereas imbalance shaped by sociocultural pressures, external ideals, or stress drives problematic patterns such as binge eating, self-induced vomiting, or restrictive dieting. Stress is particularly relevant in this interplay, as heightened perceived stress undermines self-regulation, increases vulnerability to emotional or uncontrolled eating, and intensifies body dissatisfaction (10, 11). Conversely, resilience-promoting factors such as body appreciation and functionality recognition are associated with adaptive eating and improved psychological well-being (10, 11). Thus, the interconnections among stress, eating behavior, and body image represent a multidimensional construct with potential implications for prevention and intervention.

These concerns are highly salient in the Middle East. Rapid sociocultural change, increasing exposure to Western beauty standards, and the rising prevalence of obesity have converged to create a challenging environment for body image and eating behaviors among youth (12). In Saudi Arabia, these dynamics intersect with unique cultural values and gender norms, producing distinct pressures for young adults (13). Disordered eating behaviors and body image concerns are increasingly documented among Saudi youth. Recent studies have reported that approximately 20–30% of adolescents and young adults exhibit disordered eating attitudes, with higher prevalence among females and university students (14). Similarly, body dissatisfaction has been reported in a substantial proportion of Saudi young adults, often exceeding 40% in some subgroups (13). These findings underscore the growing public health burden and the need for more nuanced, person-centered approaches to identify at-risk subgroups. University students, in particular, are at heightened risk: they face the dual challenges of academic stress and sociocultural expectations during a transitional life stage, making them especially vulnerable to stress-related eating behaviors and negative body image (10). Despite this, empirical research in the Kingdom on the intersection of stress, eating, and body image remains limited.

A central limitation of much existing research lies in its reliance on variable-centered approaches, which examine associations between single constructs in isolation. Such methods risk oversimplifying the heterogeneity of disordered eating, stress responses, and body image concerns (15). In reality, these phenomena rarely occur in uniform ways; instead, individuals often exhibit complex patterns of co-occurring symptoms and experiences. For instance, some may present with high stress but relatively adaptive eating, whereas others may show low stress yet elevated body dissatisfaction and maladaptive eating. Variable-centered methods struggle to capture such heterogeneity, potentially limiting their utility for informing targeted interventions (15).

Latent class analysis (LCA) provides a powerful alternative. It is a person-centered statistical method that identifies subgroups of individuals who share similar patterns across multiple dimensions, offering a detailed understanding of heterogeneity within a population (16). Rather than assuming uniform associations between stress, body image, and eating, LCA empirically uncovers distinct phenotypes that represent real-world variation. Prior studies in Western contexts have demonstrated the utility of LCA in identifying meaningful subtypes of eating disorders, body image profiles, and stress-related eating patterns, with implications for tailoring interventions (17, 18). To our knowledge, and based on a targeted review of the literature, few studies have applied latent class analysis to examine the co-occurrence of stress, eating behavior, and body image among university students in Saudi Arabia. Against this setting, we conducted a survey among students at the Saudi Electronic University to examine how stress, body image, and eating behaviors cluster within this population. Using validated instruments including the Perceived Stress Scale (PSS-10), the Eating Attitudes Test (EAT-26), and the Body Shape Questionnaire (BSQ-8) we applied latent class analysis to identify distinct subgroups that reflect the heterogeneity of these experiences.

This study aimed to identify distinct phenotypes among Saudi university students based on perceived stress, disordered eating behaviors, and body-image dissatisfaction. In addition, it sought to characterize these latent classes in relation to demographic factors such as age, sex, and body mass index (BMI), and to explore potential pathways for intervention by differentiating high-risk profiles from those that are subclinical. By clarifying how stress, body image, and eating behaviors intersect in this population, the study advances understanding of vulnerable groups and provides a foundation for culturally tailored prevention strategies. In doing so, it contributes not only to the scientific literature on disordered eating and body image but also to a deeper understanding of how these behaviors interrelate in the Saudi context.

## Materials and methods

### Study design and setting

This was a cross-sectional survey study conducted among undergraduate and graduate students enrolled at Saudi Electronic University campuses (SEU) across Saudi Arabia. The university is a national public institution offering blended online and in-person education across 15 regional campuses in Saudi Arabia including Dammam, Alqassim, Riyadh, Abha, Tabuk, Madinah, Alahsa, Jeddah, Jazan, Aljubail, Yanbu, Qurayyat, Alula, Hail, and Najran (19). The research team collected data using an online survey through the university’s secure electronic platform between February 20, 2025, and March 14, 2025.

### Participants

At the time of data collection, approximately 25,000 students were enrolled at the Saudi Electronic University (SEU) across all campuses. All currently enrolled SEU students were eligible to participate in the study. Participants were recruited through official institutional email invitations. Inclusion criteria required participants to be 18 years of age or older, currently enrolled at SEU, and to have provided complete responses to the main study instruments: the Perceived Stress Scale, the Eating Attitudes Test, and the Body Shape Questionnaire. Responses with missing demographic or outcome data were excluded from the final analysis. Although convenience sampling was employed, the final sample comprised 771 students representing all 15 SEU branches and a range of academic programs. Participant ages in the final sample ranged from 18 to 49 years.

### Measures

- Perceived Stress Scale (PSS-10) (20): A validated 10-item self-report instrument measuring perceived stress over the past month, with scores ranging from 0 to 40; higher scores indicate greater perceived stress. Cronbach’s *α* = 0.87
- Eating Attitudes Test (EAT-26) (21): A 26-item standardized measure assessing disordered eating attitudes and behaviors, scored from 0 to 78; a score ≥20 is commonly used as a cutoff for elevated eating concerns. In this sample, scores ranged from 0 to 66. Cronbach’s *α* = 0.89
- Body Shape Questionnaire (BSQ-8) (22): A validated scale measuring body-image dissatisfaction. The short form was used, with scores in this dataset ranging from 8 to 48; higher scores indicate greater dissatisfaction with body shape. Cronbach’s *α* = 0.79. The BSQ-8 was analyzed as a continuous measure in the latent class modeling; therefore, standard categorical cutt-off scores were not applied.
- Demographics: Age (continuous), sex (male/female), marital status (married/unmarried), and body mass index (BMI, kg/m^2^) were self-reported.

### Sample size calculation

The required sample size was estimated based on anticipated prevalence of disordered eating behaviors of 25% from prior regional studies (14). With 95% confidence level and 5% margin of error. This yielded a minimum required sample size of 451 participants. To account for potential non-response and ensure sufficient power for multivariable analysis, a 20% oversampling was applied, resulting in a target of approximately 565 participants. The final achieved sample size of 771 exceeded this requirement. All sample size estimations were performed using OpenEpi version 3.01 (Emory University, Atlanta, GA) (23).

### Sampling and recruitment

The study employed a convenience sampling method. The survey link was distributed via official SEU student email lists across all 15 campuses, targeting currently enrolled students aged 18 years and older. Multiple reminder emails were sent to encourage participation during the three-week data collection period. A total of 771 students completed the survey. Because the exact number of students who received and opened the survey invitation could not be determined, a formal response rate was not calculated. This limitation also precludes direct assessment of non-response bias. Participation was anonymous and voluntary, with no incentives offered.

### Survey piloting (internal validity)

Although the Arabic EAT-26 has been previously validated, minor linguistic adjustments were made following pilot testing to ensure clarity for Saudi university students without altering the meaning of items. Prior to data collection, the Arabic version of the questionnaire was pilot tested among 20 students representing both sexes, different academic levels, and campuses. The pilot aimed to evaluate clarity, cultural appropriateness, and ease of comprehension. Structured feedback was solicited on item wording, relevance, and clarity. Minor linguistic revisions were made to improve readability and differentiate Likert-scale responses, while technical and medical terms were simplified to ensure cultural and age-appropriate comprehension. No major content revisions were required. The refined version was subsequently used in the full survey.

### Handling of missing data

All surveys were reviewed for completeness. Questionnaires with >20% missing responses in core sections (PSS-10, EAT-26, BSQ-8) were excluded. For scales, prorated scores were calculated when at least 80% of items were answered. Missing demographic variables (e.g., BMI, marital status) were infrequent (<5%) and handled using complete-case analysis. Data were screened for improbable or implausible values (e.g., BMI < 12 kg/m^2^ or >65 kg/m^2^). Outliers were retained unless confirmed as data-entry errors, in which case corrections were applied. Data cleaning and integrity checks were conducted using SAS version 9.4. Additional checks were conducted to identify potential entry errors, such as reversed height and weight units or misplaced decimal points. Implausible cases were corrected when possible or excluded if unverifiable. Despite these procedures, BMI values remained right-skewed due to a small number of extreme but plausible values; therefore, BMI was summarized using both mean (SD) and median (IQR) to provide a more accurate representation of central tendency.

### Ethical considerations

The study protocol was approved by the Saudi Electronic University Research Ethics Committee (SEUREC-4526). All procedures followed the ethical principles of the Declaration of Helsinki. Informed consent was obtained electronically prior to participation. Responses were anonymous, and no identifying information was collected to ensure confidentiality.

### Statistical analysis

The authors analyzed the survey responses to identify unobserved subgroups of students characterized by patterns of stress, eating attitudes, and body-image concerns. Latent class analysis (LCA) was performed using total scores from the Perceived Stress Scale (PSS-10), the Eating Attitudes Test (EAT-26), and the Body Shape Questionnaire (BSQ). Gaussian mixture modeling (GMM) was selected because the primary indicators (PSS-10, EAT-26, BSQ-8) are continuous variables. Unlike traditional latent class analysis, which is designed for categorical indicators, GMM allows modeling of continuous data without loss of information due to categorization. The use of full covariance structures allows within-class correlations among indicators, partially relaxing the assumption of local independence. Models specifying 2 through 5 classes were estimated with Gaussian mixture modeling and full covariance structures (24). The optimal solution was determined on the basis of the Bayesian Information Criterion (BIC), model parsimony, and substantive interpretability (25). A 4-class solution was selected as the best fit to the data.

After class assignment, demographic characteristics were examined across classes. Age and body mass index (BMI) were compared using analysis of variance (ANOVA) with *post hoc* tests, while sex and marital status were compared using χ^2^ tests. The distributions of continuous variables (age and BMI) were assessed using visual inspection of histograms and Q–Q plots. Age was approximately normally distributed across latent classes. BMI showed mild right skewness; however, given the large sample size and the robustness of ANOVA to moderate deviations from normality, ANOVA was considered appropriate.

To further assess independent associations between demographic factors and class membership, multinomial logistic regression was conducted with class 0 (Body Image Concerned-High Eating Concerns) specified as the reference category. Odds ratios (ORs) with 95% CIs and *p* values were reported. Model assumptions were examined prior to estimation: the independence of irrelevant alternatives assumption was evaluated conceptually and deemed appropriate given the mutually exclusive nature of the latent classes; multicollinearity among predictors (age, sex, BMI, marital status) was assessed using variance inflation factors, with all values <2 indicating acceptable independence; and linearity in the logit for continuous predictors (age, BMI) was assessed by visual inspection of partial residual plots and found to be reasonable. Correlation analyses were additionally performed between class membership and demographic variables, and results were visualized using heatmaps and forest plots to facilitate interpretation. All analyses were conducted in Python, using scikit-learn for mixture modeling, stats models for regression, and seaborn/matplotlib for visualization. A 2-sided *α* of 0.05 was considered statistically significant.

Model classification quality was evaluated using entropy and average posterior probabilities (APP). Entropy was calculated to quantify overall class separation, with values closer to 1.0 indicating clearer delineation between latent classes. Class-specific APP values were computed as the mean posterior probability of assignment within each class, with values ≥0.70 considered indicative of acceptable classification accuracy.

## Results

Table 1 shows the demographic distribution of participants for the total population (N = 771) and across the four latent classes. For the total population, the mean age of the study participants was 25.1 years (SD, 6.3). The mean body mass index (BMI) was 24.94 (SD, 12.7). Of the total sample, 537 students (69.7%) were female, and 234 (30.4%) were male. With respect to marital status, 636 students (82.5%) were unmarried, whereas 135 (17.5%) were married. The mean age of participants was similar across classes, ranging from 22.6 years (SD 4.8) in Class 2 to 25.7 years (SD 6.8) in Class 1. BMI varied more substantially, with the highest mean in Class 0 (28.8, SD 7.0) and the lowest in Class 1 (22.4, SD 5.1). Women predominated in all classes, particularly in Class 2 (90.8%), whereas men were more evenly represented in Class 1 (35.4%) and Class 3 (35.5%). Most participants were unmarried, with the greatest proportion in Class 2 (90.8%) and the lowest in Class 1 (77.2%). Married participants accounted for 12.5% of Class 0 and approximately one fifth of Class 1 (22.8%) and Class 3 (18.4%).

**Table 1:** Demographic characteristics of participants across latent classes (*N* = 771).

Variable	Total population	Class 0 (*n* = 176) 22.8%	Class 1 (*n* = 237) 30.7%	Class 2 (*n* = 76) 9.9%	Class 3 (*n* = 282) 36.6%	*p*-value
Age, mean (SD)	25.08 ± (6.32)	24.6 (5.7)	25.7 (6.8)	22.6 (4.8)	25.6 (6.5)	0.001
BMI, mean (SD)	24.94 ± (3.68)	28.8 (7.0)	22.4 (5.1)	25.7 (8.7)	24.4 (6.8)	0.0001
Sex						
Female, *n* (%)	537 (69.65)	133 (75.6%)	153 (64.6%)	69 (90.8%)	182 (64.5%)	0.0001
Male, *n* (%)	234 (30.35%)	43 (24.4%)	84 (35.4%)	7 (9.2%)	100 (35.5%)	
Marital status						
Married, *n* (%)	135 (17.51%)	22 (12.5%)	54 (22.8%)	7 (9.2%)	52 (18.4%)	0.01
Unmarried, *n* (%)	636 (82.49%)	154 (87.5%)	183 (77.2%)	69 (90.8%)	230 (81.6%)	

ANOVA for continuous variables (Age, BMI).

Chi-square tests for categorical variables (Sex, Marital status).

Class 0 = Body Image Concerned –High Eating Concerns; Class 1 = High stress–body image concerned; Class 2 = Behaviorally At-risk, low body image concern; Class 3 = Moderate stress–body image vulnerable.

### Latent class analysis profiles

**Figure 1:**
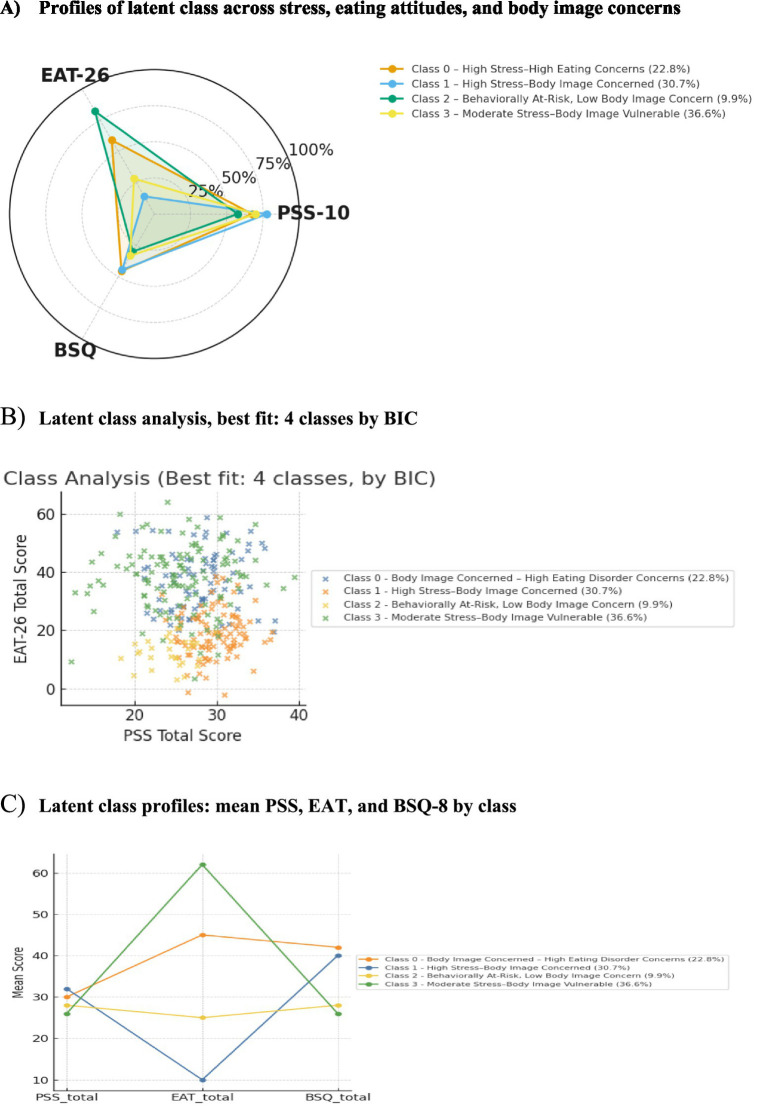
Latent class analysis profiles. **(A)** Profiles of latent class across stress, eating attitudes, and body image concerns. **(B)** Latent class analysis, best fit: 4 classes by BIC. **(C)** Latent class profiles: mean PSS, EAT, and BSQ-8 by class.

Table 2, and Figure 1A show standardized mean scores and percentages of students across stress (PSS-10), eating attitudes (EAT-26), and body-shape dissatisfaction (BSQ-8). Latent class analysis of 771 participants, using PSS-10, EAT-26, and BSQ-8 total scores, supported a four-class solution based on Bayesian Information Criterion. The 4-class solution demonstrated good classification quality, with an entropy of 0.84, indicating clear separation between latent classes. Average posterior probabilities were high across classes (Class 0: 0.88; Class 1: 0.91; Class 2: 0.86; Class 3: 0.89), supporting robust assignment of individuals to their most likely class. The classes were heterogeneous in their profiles of stress, eating attitudes, and body-image concerns. Class 0 (*n* = 176; 22·8%) was characterized by moderate stress (mean 27 [67·5% of maximum]), high eating concerns (46 [59·0%]), and moderate to high body-image dissatisfaction (41 [45·6%]), and was labeled Body Image Concerned –High Eating Concerns. Class 1 (*n* = 237; 30·7%) demonstrated high stress (31 [77·5%]) and marked body-image concerns (40 [44·4%]), but low eating-attitude disturbance (11 [14·1%]), and was labeled High Stress–Body Image Concerned. Class 2 (n = 76; 9·9%) was distinguished by elevated eating concerns (64 [82·1%]) despite modest stress (23 [57·5%]) and low body-image dissatisfaction (27 [30·0%]), representing a Behaviorally At-risk, Low Body Image Concern profile. Class 3 (*n* = 282; 36·6%) reflected moderate to high stress (28 [70·0%]), modest eating concerns (22 [28·2%]), and high body-image vulnerability (30 [33·3%]), and was labeled Moderate Stress–Body Image Vulnerable.

**Table 2:** Latent class profiles across stress, eating attitudes, and body-image concerns.

Class	*n* (%)	PSS-10 Mean (%, of max)	EAT-26 Mean (%, of max)	BSQ-8 Mean (%, of max)	Profile label
Class 0	176 (22.8%)	27 (67.5%)	46 (59.0%)	41 (45.6%)	Body Image Concerned –High Eating Concerns
Class 1	237 (30.7%)	31 (77.5%)	11 (14.1%)	40 (44.4%)	High stress–body image concerned
Class 2	76 (9.9%)	23 (57.5%)	64 (82.1%)	27 (30.0%)	Behaviorally at-risk, low body image concern
Class 3	282 (36.6%)	28 (70.0%)	22 (28.2%)	30 (33.3%)	Moderate stress–body image vulnerable

Figure 1C illustrates the mean raw scores for PSS-10, EAT-26, and BSQ-8 across classes. Results mirrored Figure 1A, confirming four distinct phenotypes: Body Image Concerned-High Eating Concerns (Class 0), high stress-body image concerned (Class 1), behaviorally at-risk, low body image concern (Class 2), and a Moderate Stress–body image vulnerable (Class 3). Together, Figures 1A–C demonstrate heterogeneous pathways: higher stress levels were associated with greater eating concerns and body dissatisfaction, while a subgroup exhibits severe eating concerns without body-image disturbance, and a large proportion remains in subclinical but vulnerable states.

Conversely, Class 1 (high stress–body image concerned) and Class 3 (moderate stress–body image vulnerable) were negatively correlated with BMI (r = −0.24 and −0.21, respectively), indicating these classes were more common among lower-BMI students. Class 2 (behaviorally at-risk, low body-image concern) had weaker associations overall but showed a slight negative correlation with BMI (r = −0.10). Sex also differentiated profiles: female students correlated positively with Class 2 (r = 0.15), consistent with their predominance in this subgroup, while negatively correlating with Class 3 (r = −0.18). Class 1 showed only a slight positive association with female sex (r = 0.07). Age was modestly correlated with marital status (r = 0.58), as expected, but only weakly associated with class membership overall. Married students showed small positive correlations with Class 1 (r = 0.20) and Class 3 (r = 0.07), while being negatively associated with Class 2 (r = −0.07).

**Figure 2:**
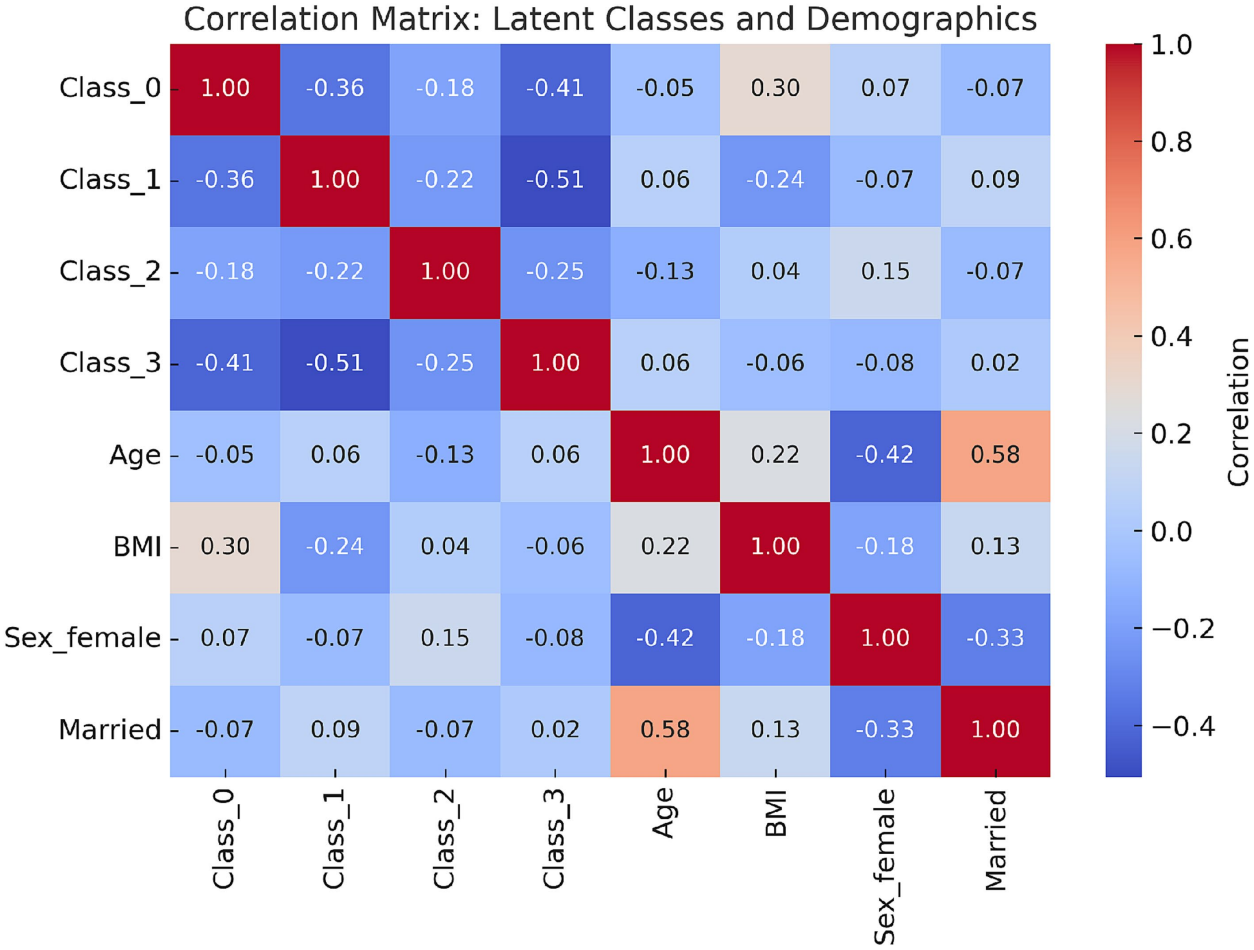
Correlation matrix: LCA classes and demographics.

The correlation matrix in Figure 2 illustrates how latent class membership relates to demographic characteristics (age, BMI, sex, and marital status). Class 0 (Body Image Concerned-High Eating Concerns) showed a modest positive correlation with BMI (r = 0.30), suggesting that students with higher BMI were more likely to fall into this group. Conversely, Class 1 (high stress–body image concerned) and Class 3 (moderate stress–body image vulnerable) were negatively correlated with BMI (r = −0.24 and −0.21, respectively), indicating these classes were more common among lower-BMI students. Class 2 (behaviorally at-risk, low body image concern) had weaker associations overall but showed a slight negative correlation with BMI (r = −0.10). Sex also differentiated profiles: female students correlated positively with Class 2 (r = 0.15), consistent with their predominance in this subgroup, while negatively correlating with Class 3 (r = −0.18). Class 1 showed only a slight positive association with female sex (r = 0.07). Age was modestly correlated with marital status (r = 0.58), as expected, but only weakly associated with class membership overall. Married students showed small positive correlations with Class 1 (r = 0.20) and Class 3 (r = 0.07), while being negatively associated with Class 2 (r = −0.07).

**Figure 3:**
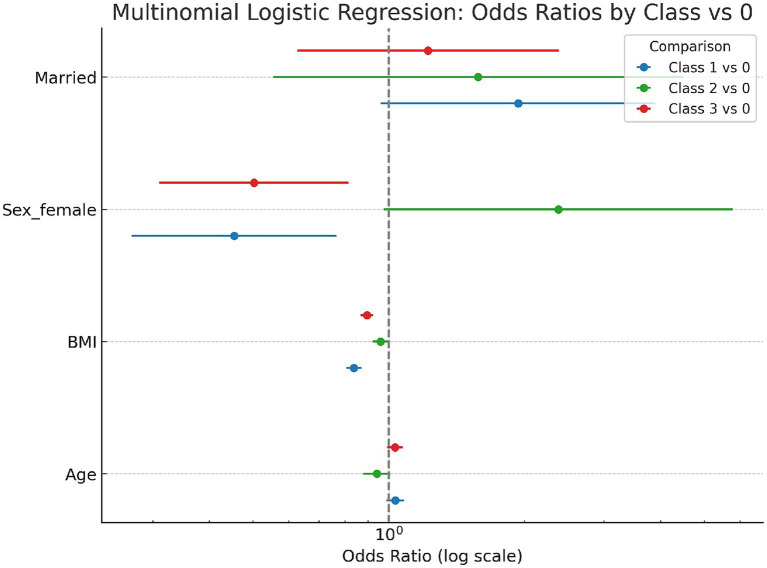
Forest plot for predictors of class 1, 2, and 3 vs. 0.

Table 3 and Figure 3 present the associations of age, sex, BMI, and marital status with latent class membership (reference = Class 0, Body Image Concerned –High Eating Concerns). BMI showed the most consistent associations across classes. Each one-unit increase in BMI was associated with significantly lower odds of membership in Class 1 (high stress–body image concerned; OR 0.84, 95% CI 0.81–0.87; *p* < 0.001) and Class 3 (Moderate Stress–Body Image Vulnerable; OR 0.89, 95% CI 0.87–0.92; *p* < 0.001), and with modestly reduced odds of Class 2 (Behaviorally At-risk, Low Body Image Concern; OR 0.96, 95% CI 0.92–1.00; *p* = 0.036). Female sex was associated with substantially lower odds of being in Class 1 (OR 0.45, 95% CI 0.27–0.77; *p* = 0.003) and Class 3 (OR 0.50, 95% CI 0.31–0.81; *p* = 0.005), but with a non-significant tendency toward higher odds of Class 2 (OR 2.38, 95% CI 0.98–5.79; *p* = 0.057). Marital status and age were not significantly associated with class membership, though married participants tended to have higher odds of belonging to Class 1 (OR 1.93, 95% CI 0.96–3.90; *p* = 0.06).

**Table 3:** Multinomial logistic regression of demographic predictors of latent class membership (reference category = Class 0, Body Image Concerned-High Eating Concern).

Predictor	Class 1 vs. 0			Class 2 vs. 0			Class 3 vs. 0		
	OR^1^	95% CI^2^	*p* value	OR	95% CI	*p* value	OR	95% CI	*p*-value
Age	1.03	(0.99–1.08)	0.142	0.94	(0.88–1.0)	0.086	1.03	(0.99–1.08)	0.122
BMI	0.84	(0.81–0.87)	<0.001	0.96	(0.92–1.00)	0.036	0.89	(0.87–0.92)	<0.001
Female vs. male	0.45	(0.27–0.77)	0.003	2.38	(0.98–5.79)	0.057	0.50	(0.31–0.81)	0.005
Married vs. unmarried	1.93	(0.96–3.90)	0.065	1.58	(0.56–4.48)	0.392	1.22	(0.63–2.39)	0.556

1 OR = Odds ratio; 2 95% Confidence interval; Class 0 = Body Image Concerned –High Eating Concerns; Class 1 = High stress–body image concerned; Class 2 = Behaviorally at-risk, low body image concern; Class 3 = Moderate stress–body image vulnerable.

## Discussion

This study identified four distinct phenotypes of stress, eating attitudes, and body-image concerns among Saudi university students. The findings underscore that the heterogeneity of pathways is associated with disordered eating and highlight the importance of person-centered approaches for prevention and early intervention. Rather than a uniform trajectory, our results reveal that students show different patterns of co-occurring stress, body-image dissatisfaction, and maladaptive eating behaviors. These insights are highly relevant in the Saudi context, where rapid sociocultural change, growing pressures around academic performance, and shifting beauty ideals converge to shape health risks in young adults (12, 13, 26).

### Heterogeneity in stress–eating–body image profiles

The four latent classes identified demonstrate two dominant pathways. The first was stress-driven, combining high perceived stress with either eating concerns (Class 0, 1) or body-image dissatisfaction. The second pathway was behaviorally driven, where elevated disordered eating attitudes occurred even in the absence of body-image concerns (Class 2). A substantial proportion of students also occupied a subclinical “moderate stress–body image vulnerable” group (Class 3), representing nearly 40% of the sample. These findings align with international research demonstrating that disordered eating is not one-dimension but instead reflects multiple psychosocial and behavioral configurations (15, 17, 18) Importantly, the presence of a behaviorally at-risk group with minimal body-image concerns suggests that conventional screening, which often emphasizes dissatisfaction with body shape, may miss individuals who nonetheless practice maladaptive eating behaviors.

### Role of BMI and sex in shaping phenotypes

Multinomial regression analyses revealed BMI and sex as key correlates of latent class membership. Students with higher BMI were more likely to belong to the high stress–high eating concerns class but less likely to fall into body-image vulnerable groups, suggesting that stress-eating linkages in higher-weight individuals may reflect more behavioral than perceptual. Conversely, students with lower BMI were disproportionately represented in body-image–dominated classes. Female sex was associated with elevated odds of membership in the behaviorally at-risk class, even when body-image dissatisfaction was low, echoing prior findings that women may adopt restrictive or maladaptive eating behaviors independent of overt dissatisfaction with body shape (2, 14). These results reinforce the need for sex-sensitive and weight-sensitive interventions. While weight management and lifestyle counseling remain important for higher-BMI students, lower-BMI and female students may require closer monitoring for subclinical or covert disordered eating behaviors.

### Implications for prevention and student health services

The high prevalence of subclinical risk in this population particularly the large Class 3 group emphasizes the need for preventive action. Interventions that normalize stress management, promote body appreciation, and encourage healthy coping strategies may help to reduce the risk of escalation into clinically significant disorders. In line with Saudi Vision 2030, which prioritizes youth health and well-being as part of broader national transformation goals, universities can play a central role in implementing culturally adapted wellness programs (27). These may include embedding stress-management workshops into orientation, developing confidential self-screening platforms, and offering integrated counseling services that address both psychosocial and behavioral health.

Importantly, prevention should not rely on a “one-size-fits-all” strategy. For students in stress-driven groups (Classes 1), interventions focusing on resilience, mindfulness, and cognitive reframing may be most effective (28). For those in the behaviorally at-risk group (Class 2), direct monitoring of eating behaviors even in the absence of body-image complaints is critical. For the group (Class 3), psychoeducation and community-based initiatives that promote positive embodiment and reduce stigma can help avert progression. Leveraging digital health platforms, which are widely accessible in Saudi Arabia, could extend the reach of such interventions to geographically dispersed students across SEU’s multiple campuses (29).

### Cultural context and relevance to Saudi Arabia

Our findings should be interpreted in light of Saudi Arabia’s unique cultural and sociocultural landscape. Prior studies have highlighted how Western beauty ideals, rapid modernization, and social media exposure intersect with local gender norms to produce distinct body-image pressures in the Kingdom (12, 13). At the same time, traditional values emphasizing modesty and family reputation may contribute to underreporting of body-image dissatisfaction, partially explaining the emergence of a class defined by eating concerns without dissatisfaction (30). The intersection of global and local cultural narratives thus creates complex dynamics that shape how students experience and respond to stress, body image, and eating behaviors. Recognizing this cultural specificity is essential for designing prevention programs that are both effective and acceptable to Saudi students.

### Comparison with existing literature

Our results parallel findings from Western contexts, where LCA has identified heterogeneous subtypes of eating disorders and stress–body image profiles (17, 18). However, the prominence of a large subclinical class and the emergence of a behaviorally driven at-risk group suggest that Saudi students may exhibit distinct risk patterns. This underscores the need for context-specific research and validates the use of person-centered methods such as LCA in Saudi populations. By moving beyond variable-centered models, which oversimplify the complexity of these phenomena, this study provides a broader understanding of disordered eating risk among university students in Saudi Arabia.

### Limitations

Several limitations warrant consideration. First, the study used a cross-sectional design, precluding causal inference regarding the directionality of associations among stress, body image, and eating concerns. Longitudinal studies are needed to examine how latent classes evolve over time and whether transitions occur between subclinical and high-risk profiles.

Second, the use of email-based convenience sampling precluded calculation of a formal response rate, as the number of students who received and opened the survey invitation was unknown. Consequently, the potential for non-response bias cannot be ruled out, and participants who chose to respond may differ systematically from those who did not. This may limit the sample’s representativeness and should be considered when interpreting the generalizability of the findings.

Third, data were derived from self-report instruments, which may be subject to recall bias, social desirability bias, and underreporting, particularly in sensitive domains such as body image and eating behavior (31). Fourth, the convenience sampling strategy, though effective in securing a large and diverse sample across SEU campuses, may limit generalizability to other Saudi university populations. Fifth, BMI was calculated based on self-reported height and weight, which may be subject to systematic reporting bias. Participants tend to underestimate weight and overestimate height, which can lead to an underestimation of BMI. This measurement error may have influenced the observed associations between BMI and latent class membership. Finally, cultural nuances in the interpretation of body-image questions may influence responses despite careful piloting and validation.

## Conclusion

This study demonstrates that Saudi university students are not a homogenous group with respect to stress, eating attitudes, and body image. Instead, they comprise multiple phenotypes, each with distinct risk factors and intervention needs. By identifying both overtly high-risk and subclinical groups, our findings support the development of stratified, culturally sensitive prevention programs aligned with Saudi Vision 2030’s commitment to youth health. Future research should focus on longitudinal validation of these classes, the integration of biological or digital biomarkers, and the testing of targeted interventions to reduce progression toward disordered eating and associated psychosocial burdens.

## Data Availability

The raw data supporting the conclusions of this article will be made available by the authors, without undue reservation.
